# The inverse association between relative abundances of oleic acid and arachidonic acid is related to alpha -linolenic acid

**DOI:** 10.1186/1476-511X-13-76

**Published:** 2014-05-10

**Authors:** Arne Torbjørn Høstmark, Anna Haug

**Affiliations:** 1 Section of Preventive Medicine and Epidemiology, University of Oslo, Norway, Institute of Health and Society, Box 1130 Blindern, 0318 Oslo, Norway; 2 Department of Animal and Aquacultural Sciences, The Norwegian University of Life Sciences, Box 5003, 1432 Ås, Norway

## Abstract

**Background:**

Many health effects of oils rich in oleic acid (OA, 18:1 n9) seem to be opposite those of arachidonic acid (AA, 20:4 n6), i.e. concerning cardiovascular risk. In recent studies in humans and in the rat we observed that percentages of OA and AA were inversely related, raising the question of whether the inverse association is a general one, and how it might be explained. In the present work we examine whether percentages of OA and AA are inversely associated in breast muscle lipids of chickens, and whether alpha-linolenic acid (ALA) may be related to the OA/AA ratio.

**Methods:**

The study group consisted of 163 chickens. Breast muscle was collected, and the concentration of fatty acids in muscle lipids was determined using gas chromatography. We studied association between fatty acids using bivariate correlations (Pearson) and linear regression. Synthesis of OA from stearic acid (Stear) was estimated using the OA/Stear ratio, and formation of AA from linoleic acid (LA) was estimated by the AA/LA ratio.

**Results:**

We found a strong inverse relationship (r = -0.942, p < 0.001; n = 163) between % OA and % AA in breast muscle lipids of the chickens. There was an inverse association (r = -0.887, p < 0.001) between the OA/Stearic acid ratio, estimating Delta9 desaturase, and the AA/LA ratio, estimating desaturases/elongase activities. Furthermore, there was a strong negative association between % AA and the OA/Stearic acid ratio (r = -0.925, p < 0.001), and % OA correlated negatively (r = -0.914, p < 0.001) with the AA/LA ratio. ALA was positively associated (r = 0.956, p < 0.001) with the OA/AA ratio, and this association prevailed when controlling for the other fatty acids. ALA was positively associated (r = 0.857, p < 0.001) with the OA/Stear ratio, but was negatively related (r = -0.827, p < 0.001) to the AA/LA ratio.

**Conclusions:**

The relative abundances of OA and AA that are inversely related in muscle lipids of chickens may be explained by a feedback regulation between the synthesis of OA and AA, and related to ALA, which seems to stimulate formation of OA, and inhibit synthesis of AA, but further studies are required to clarify whether this hypothesis is valid.

## Background

It is widely accepted that oleic acid (OA, 18:1 n9), and oleic acid rich foods such as olive oil may have many beneficial health effects. Among such effects are improved insulin sensitivity, and endothelium-dependent flow-mediated vasodilatation [1], lowering of LDL cholesterol [2,3] and an increase in HDL cholesterol [4]. If lipids in LDL are enriched in oleic acid, the particles will be less liable to be oxidized [5], a property that is of significance for the normal metabolism of LDL [6]. Furthermore, intake of oleic acid seems to be associated with reduced blood pressure [7]. Thus, many of the effects of oleic acid may serve to reduce the risk of cardiovascular diseases. Additionally, the fatty acid may have anti-carcinogenic and anti-inflammatory effects [8-10].

Although beneficial effects of oils rich in oleic acid have been reported, the mechanisms by which such oils might have beneficial health effects are still incompletely understood. Various antioxidants present in e.g. virgin olive oil, as well as the high content of oleic acid, could partly explain the health effects.

When considering the reported beneficial health effects of oils rich in oleic acid, we previously suggested [11,12] that many of the positive effects would be anticipated if the fatty acid works to counteract effects of arachidonic acid (AA, 20:4 n6). This fatty acid is formed in the body from linoleic acid (LA, 18:2 n6), a major constituent in many plant oils, and is converted by cyclooxygenase and lipoxygenase into various eicosanoids, i.e. prostacyclines, thromboxanes and leukotrienes [13]. AA derived thromboxane A_2_ (TXA_2_) and leukotriene B_4_ have strong proinflammatory and prothrombotic properties [14,15]. Furthermore, endocannabinoides, which are derived from arachidonic acid, may have a role in adiposity and inflammation [16].

An interaction between oleic acid and arachidonic acid was suggested several decades ago in the rat [17]. More recently, Cicero et al. [5] showed in human subjects that supplementation with a high dose of olive oil for 3 weeks resulted in an increase in LDL oleic acid and a decrease in linoleic and arachidonic acid. Also in chicken breast muscle a negative 18:1 n9 vs. 20:4 n6 association was observed [18].

One mechanism by which OA could counteract those of AA is to reduce the relative abundance of AA in serum and tissues. Conceivably, increased supply of oleic acid might reduce that of AA by pure mass action. Inverse regulation could also be effected through more specific metabolic feedback regulation. For example, a reduced percentage of AA would be expected if OA inhibits Delta-6 desaturase, Elongase-5 (Elovl-5) and/or Delta-5 desaturase, the enzymes governing formation of AA from LA. Conversely, inhibition by AA of Delta-9 desaturase should lower percentage OA, and previous studies suggest that this latter mechanism might take place [19].

It seems that the Delta-9 desaturases are of considerable physiological significance. Thus, regulation of the amount of monounsaturated fatty acids (MUFA) has the potential to affect a variety of key physiological variables, such as insulin sensitivity, metabolic rate, adiposity, atherosclerosis, cancer and obesity [19,20].

In accordance with the above considerations, in a rat study we recently reported that percentages of OA and AA were inversely related [11]. Subsequently, we observed a similar inverse association in the phospholipid faction of human sera [12]. These observations raise the question of whether the OA vs. AA relationship is a general one. In the present work we use data obtained in a previous study [21] to extend our previous work by examining whether an inverse OA vs. AA association might also exist in chickens.

Although a direct feedback regulation between the synthesis of OA and AA seems to be involved to explain the appreciable variation in the OA/AA ratio, the possibility exists that this ratio is also governed by other fatty acids. One particular candidate is ALA, the precursor of the endogenous synthesis of EPA, and known to have many health effects [22-24]. Therefore, as part of the present work we included analyses to examine whether ALA might be related to the OA/AA ratio.

## Results

### Is there a direct feedback regulation between the synthesis of OA and AA in lipids of chicken breast muscle?

**Figure 1:**
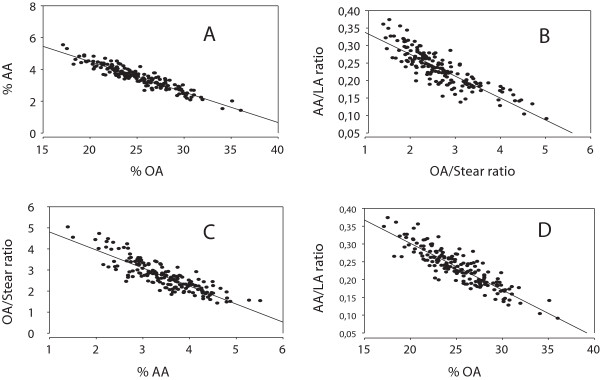
**Relationship between estimates of OA and AA formation.** Relationship between relative amounts of OA and AA (Panel **A**) in breast muscle lipids of chickens fed various diets containing linseed oil, pooled data analysis (see Methods). Panel **B**: association between estimates of OA and AA synthesis, i.e. product/precursor ratios. Panel **C**: Relationship between % AA and product/precursor estimate of OA synthesis. Panel **D**: relationship between % OA and product/precursor estimate of AA formation from LA. OA = oleic acid (18:1 n9); Stear = stearic acid (18:0); AA = arachidonic acid (20:4 n6); LA = linoleic acid (18:2 n6). Note broken axes. Coefficients of correlation in panels **A**, **B**, **C**, and **D** were: -0.936 (p < 0.001), -0.856 (p < 0.001), -0.859, p < 0.001), and -0.884, p < 0.001), respectively. When analyzing the same relationships in each of the diet subgroups (see Methods) separately, we obtained highly significant correlation coefficients (r > 0.8; p < 0.001) in all subgroups.

We found a tight inverse relationship (r = -0.936, p < 0.001; n = 163) between percentages of OA and AA in breast muscle lipids of the chickens (Figure 1, panel A). There was also a strong inverse relationship between the product/precursor index estimating synthesis of OA from stearic acid, and the product/precursor estimate of AA synthesis from LA (r = -856, p < 0.001, Figure 1, panel B). In addition, we found strong inverse associations between % AA and the OA/Stearic acid ratio, an estimate of OA synthesis (r = -0. 859, p < 0.001, panel C), and also between % AA and the AA/LA ratio, estimating AA synthesis from LA (r = -0.884, p < 0.001, panel D). Diet type did not influence the association; when analyzing the relationships shown in Figure 1 in each diet subgroup separately (see Methods), we obtained highly significant correlations (r > 0.8; in many groups: r > 0.9); with p < 0.001 in all subgroups (results not shown otherwise).

### Is the variation in the OA/AA ratio related to ALA?

**Figure 2:**
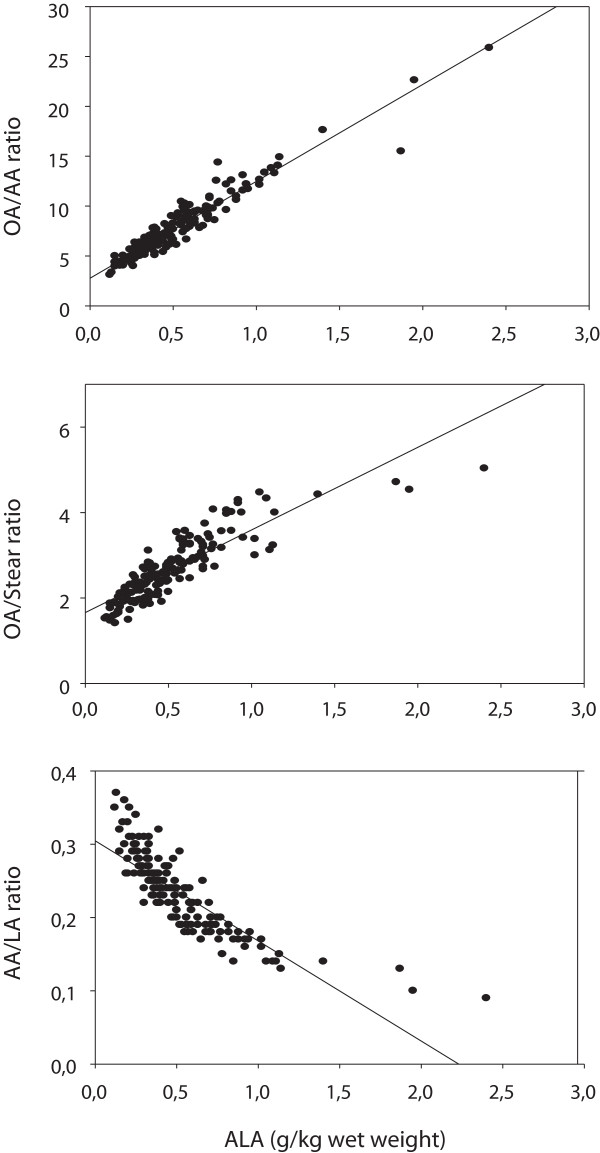
**Relationship between ALA and the OA/AA ratio (top panel), the OA/Stear ratio (middle panel), and the AA/LA ratio (lower panel), in breast muscle phospholipids of chickens fed various diets, pooled data analysis (see Methods).** ALA = alpha linolenic acid (18:3 n3); OA = oleic acid (18:1 n9); Stear = stearic acid (18:0); AA = arachidonic acid (20:4 n6); LA = linoleic acid (18:2 n6). Coefficients of correlation in the top, middle, and lower panels were: 0.956 (p < 0.001), 0.857 (p < 0.001), and -0.827 (p < 0.001), respectively. When analyzing the same relationships in each of the diet subgroups separately (see Methods), we obtained highly significant correlation coefficients (r > 0.8; p < 0.001) in all subgroups.

The results shown in Figure 1 imply a considerable variation in the OA/AA ratio in breast muscle lipids of the chickens. As shown in Figure 2, top panel, there was a strong positive association between ALA and the OA/AA ratio (r = 0.956, p < 0.001, n = 163).

Using multiple linear regression we next studied whether the ALA vs. OA/AA ratio association would persist when controlling for other fatty acids. As shown in Table 1, ALA was still significantly (p < 0.001) associated with the OA/AA ratio when controlling for diet type and all of the other fatty acids measured (Model 4, Table 1). As judged from the magnitude of the standardized regression coefficient, the association was weakened in the adjusted model.

**Table 1:** Association between ALA (independent variable under investigation) and the OA/AA ratio (dependent variable), multiple linear regression

**Model**	**B (SE)**	**Beta**	**t**	**p**
1	9.71 (0.23)	0.956	41.5	<0.001
2	9.49 (0.22)	0.934	42.3	<0.001
3	12.50 (0.79)	1.231	15.9	<0.001
4	8.42 (1.43)	0.828	5.9	<0.001

Dependent variable = OA/AA ratio. B = unstandardized regression coefficient, Beta = standardized coefficient. Model 1 is without adjustment; Model 2: adjusted for 20:3 n3 + 20:5 n3 + 22:5 n3 + 22:6 n3 ; Model 3 = Model 2 + 18:2 n6 + 18:3 n6 + 20:2 n6 + 20:3 n6; Model 4 = Model 3 + 14:0 + 14:1 c9 + 15:0 + 16:0 + 16:1 c9 + 17:0 + 18:0 + 18:2 c11 + 18:2 n6 + 20:0 + 20:1 n9 + 20:2 n6 + 20:3 n6 + unidentified 18:1 isomers ( minor amounts) + diet type.

### Is ALA a stimulator of OA synthesis and/or an inhibitor of AA synthesis?

A positive relationship between ALA and the OA/AA ratio (Figure 2, upper panel) would be expected if ALA is a stimulator of the synthesis of OA and/or an inhibitor of the synthesis of AA. We therefore studied the association between ALA and the synthesis of OA and AA, as assessed by the product/precursor ratio. Indeed, there was a highly significant positive association (r = 0.857, p < 0.001) between ALA and the OA/Stear ratio, estimating OA synthesis (Figure 2, middle panel). Furthermore, we observed a negative association (r = -0.827, p < 0.001) between ALA and the AA/LA ratio, estimating AA synthesis from LA (Figure 2, lower panel). Multiple linear regression analyses with control for diet type and all of the other fatty acids showed that these associations prevailed (ALA vs. OA/Stear: t = 6.2, p < 0.001; ALA vs AA/LA: t = -2.4, p = 0.021, results not shown otherwise). When analyzing the associations shown in Figure 2 in each diet subgroup separately (see Methods), we obtained highly significant (p < 0.001) positive and negative, respectively, correlations (r > 0.8; in many subgroups r > 0.9) in all subgroups (results not shown otherwise).

## Discussion

The present work indicates that there is a close inverse relationship between percentages of OA and AA acid in breast muscle lipids of chickens. The result supports our previous reports showing a similar inverse relationship between % OA and % AA in serum phospholipids of young, healthy subjects [12], and also found in total serum lipids of rats [11] It would appear, therefore, that the observed inverse % OA vs. % AA relationship is a general one.

In the rat, an interaction between OA and linoleic acid, the precursor of AA, was reported several decades ago [17], and in a multi-center randomized cross-over study involving 200 healthy European subjects, Cicero et al. more recently [5] showed that a 3 weeks supplementation with olive oil resulted in an increase in OA in LDL and a decrease in LA and AA. The increase in the OA/LA ratio was accompanied by reduced levels of isoprostanes, biomarkers of oxidative stress.

The finding of a negative correlation between OA and AA also in mice treated with perfluorinated fatty acids [25], zenobiotics used as surfactants in various industrial products, would seem in support of the contention that the inverse relationship between this couple of fatty acids exists in many species.

By pure mass action, increased supply of OA might replace AA in various compartments, such as in the lipids of cell membranes. Conceivably, increase in the percentage of one particular fatty acid must be accompanied by a reduction in the percentage of one or more of the other fatty acids. This type of mechanism was previously suggested by the results of a previous diet trial in chicken [18].

It has been suggested that OA is a weak competitive inhibitor of cyclooxygenase [26], which catalyzes conversion of the C20 PUFAs AA and EPA into prostaglandins, thromboxanes and leucotrienes [13], and OA might accordingly increase AA and EPA.

Another mechanism serving to explain the inverse relationship could be that OA acts as an inhibitor of Delta-5/6 desaturases and/or Elongase-5 so as to reduce the formation of AA. Additionally, the possibility exists that AA might inhibit the formation of OA by inhibition of Delta-9 desaturase. Possibly, inhibition by AA of Delta-9 desaturase gene transcription might be involved, since previous studies suggest that PUFAs of both the n6 and n3 families can inhibit this transcription [20]. The inverse association between % OA and the AA/LA ratio, used as an estimate of AA synthesis from LA, the inverse association between % AA and the OA/Stear ratio, estimating OA synthesis, as well as the inverse association between the ratios estimating synthesis of OA and AA, respectively, i.e. the OA/Stear ratio and the AA/LA ratio, appear to be in accordance with this hypothesis.

The linear inverse association between relative abundances of OA and AA in chicken muscle lipids indicates that the OA/AA ratio may vary appreciably. Furthermore, the present multiple regression analyses suggest that ALA might at least partly govern the ratio between OA and AA in muscle lipids of chickens. The association between ALA and the OA/AA ratio prevailed with high significance when controlling for each of the several fatty acids measured. The association was attenuated when controlling for all of the fatty acids measured (Table 1, regression Model 4), as judged by the magnitude of the standardized regression coefficient. However, in this case we might have an overadjustment bias [27], due to control for some fatty acids which are intermediate variables (or a descending proxy for an intermediate variable) on a causal path from exposure to outcome. One example is LA which is the precursor of AA, a component of the outcome variable. On the other hand, some of the fatty acids entered among the independents may not solely be intermediates, but also serve as regulators of enzymes involved in the metabolism of fatty acids in both the n3 and n6 families. Since the regulatory function of fatty acids is not completely clarified, we have included all of the measured fatty acids in our regression analyses. Although the highly significant positive association between ALA and the OA/AA ratio prevailed also when including other fatty acids in the regression model, we cannot rule out the possibility that our results might be explained by as yet unknown covariates, and by the several inter-correlations among the fatty acids.

The observed tight positive coupling between ALA and the product/precursor estimate used for OA synthesis, as well as the strong negative association between ALA and the AA/LA ratio used to estimate AA synthesis from LA, seem to be in favor of this hypothesis. It would appear, accordingly, that one interpretation of the present results could be that the inverse % OA vs. % AA relationship is attributed not only to a direct feedback regulation between the synthesis of this couple of fatty acids, but that ALA, and possibly other fatty acids, may participate to govern the % OA vs. % AA relationship. Also our preliminary results obtained in total lipids of sera from 36 male rats suggest that ALA is positively correlated with the OA/Stear ratio (r = 0.463, p = 0.004), and negatively with the AA/LA ratio (r = -0.551, p < 0.001; results not published). Thus, one explanation of the finding that ALA was positively associated with the OA/AA ratio could be that ALA acts as a stimulator of OA synthesis and /or as an inhibitor of AA synthesis.

On the other hand, there are previous reports which apparently do not seem in support of our findings. Thus, in general, PUFAs like ALA may decrease rather than stimulate stearoyl-CoA desaturase (SCD1) expression and activity [28-32]. From these previous results we might have expected ALA to be inversely - rather than positively - related to the OA/AA and the OA/stearic acid ratios. Apparent discrepancies could possibly be related to differences in approach, for example concerning diet and species used, sex, and whether the study was done in vitro or in vivo. In any instance, the n3 PUFAs including ALA have many functions, and also as yet unknown mechanisms might be involved in their regulation.

We emphasize that the product/precursor ratio used in the present study are only crude estimates of enzyme activities, and more direct methods are needed to clarify whether the suggested mechanisms are valid.

It would appear that many of the alleged positive health effects of OA should be expected if OA acts to counteract effects of, or reduces the relative amounts of AA. Furthermore, health effects of ALA have been attributed to the fact that this fatty acid can be converted to EPA [23]. The associations with ALA studied in the present work do not seem, however, to be explained by a similar mechanism. Since the present analyses are based upon data from a diet trial in chickens, we will not discuss possible health implications of the findings.

The present analyses were carried out on pooled data from a previously published diet trial involving several subgroups [21]. The diets used for the present study contained 2.4% linseed oil and had equal n6/n3 ratio, but differed in amount of rendered fat, palm oil, red palm oil, and rapeseed oil. Furthermore, in some subgroups the diets had increased amount of selenium [21]. Thus, there was a 2-fold variation between diets in the content of palmitic acid, and a 3-fold variation in the content of stearic acid. However, in spite of appreciable differences among the diets, the slope of the association curve between ALA and the OA/AA ratio remained unaffected of the diet. For example, a 2-fold increase in the intake of palmitic acid did not influence the OA vs. AA relationship.

We suggest that the consistent inverse relationship between percentages of OA and AA, possibly governed both by a direct feedback regulation between formation of OA and AA, and also influenced by ALA, could possibly be related to the risk of AA associated conditions and diseases, such as inflammation and cardiovascular diseases. However, the present data are not sufficient to substantiate this hypothesis, and further studies in man are required to examine the possibility. Whatever the mechanisms might be, the present results support our previous observation that percentages of oleic acid and arachidonic acid are inversely related.

## Conclusions

**Figure 3:**
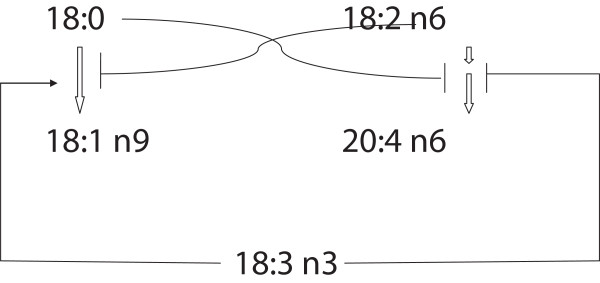
**A hypothesis suggested to explain the inverse association between relative abundances of OA and AA in chicken breast muscle lipids, based upon our previous and the present results.** According to the hypothesis, OA may inhibit synthesis of AA from LA (inverse relationship between % OA and the AA/LA ratio). Conversely, AA may inhibit formation of OA from stearic acid (inverse relationship between % AA and the OA/Stearic acid ratio, the product/precursor estimate of Delta9 desaturase). In addition, the OA/AA ratio may be governed by ALA, which may stimulate OA formation (positive association between ALA and the OA/Stearic acid ratio), and inhibit synthesis of AA from LA (negative association between ALA and the AA/LA ratio).

The relative abundances of OA and AA in lipids of chicken breast muscle are inversely related. The results are in agreement with previous findings in man and in the rat, raising the question of whether this relationship is a general one across various species. The results suggest that there might be an inverse coordinated regulation of the formation of the two fatty acids, possibly effected by a feedback regulation between the synthesis of OA and AA, and possibly related to ALA (see Figure 3), but further studies are required to clarify whether the hypothesis is valid.

## Methods

The present study is a spin-off of a previously published diet trial [21], in which groups of chickens were fed different types of diet.

### Ethical approval

The diet trial in chickens was performed in accordance with National and international guidelines concerning the use of animals in research (Norwegian Animal and Welfare Act, European Convention for the protection of Vertebrate Animals used for Experimental and other Scientific Purposes, CETS No.: 123 1986). The trial was approved by the Regional Norwegian Ethics Committee, and the experimental research followed internationally recognized guidelines.

### Chickens and diet

One day old Ross 308 broiler chickens from Samvirkekylling (Norway) were randomly divided into 10 groups with about 16 birds in each group, and fed equienergetic wheat based diets, from day 1 to 29. The animals had free access to feed and water. They were kept group wise in mesh floored battery cages from day 1 to day 12, and then placed in separate metabolism cages from day 12 until day 29. The birds were inspected twice daily by qualified handlers, and every other day by a veterinarian throughout the trial period. The feeds were all containing 8% supplement fat, of which 2.4% was linseed oil. The n6/n3 ratio of the diets was similar, being about 1.5. Content of rendered fat, palm oil, red palm oil and rapeseed oil varied as shown in Table 2. The various diets had identical amounts of the various ingredients, except for the type of fat. In addition, in five subgroups extra selenium was added [21]. Since our preliminary analyses showed that the various diets did not influence the associations studied in the present work, only the results of the pooled sample are presented in detail.

**Table 2:** Fatty acid composition (%) of individual diets (see comment below)

	**C16:0**	**C18:0**	**C18:1 c9**	**C18:2 n6**	**C18:3 n3**	**n6/n3**
2.4% LO + 5.6% RF	18.1	10.1	28.0	19.8	14.3	1.4
2.4% LO + 5.6% PO	27.9	3.3	30.2	21.7	13.7	1.6
2.4% LO + 5.6% RPO	28.5	3.5	29.5	21.9	13.6	1.6
2.4% O + 1.6% RO + 4% RF	15.3	7.9	31.5	21.4	15.9	1.4
2.4% LO + 1.6% RO + 4% PO	22.2	2.9	32.8	23.2	15.5	1.5

LO = linseed oil; RF = rendered fat; PO = palm oil; RPO = red palm oil; RO = rapeseed oil.

Note: Since all diets gave the same outcome concerning the present statistical analyses, we only present results of the pooled data in detail.

At day 29, the animals were stunned by a sharp blow to the head and killed by exsanguination. Samples from the breast muscle were frozen at -20°C for fatty acid analyses as described by O’Fallon et al. [33].

### Determination of fatty acids

Fatty acid composition of total lipids of breast muscle and feed was determined by gas chromatography [29]. Fatty acid content is presented as g fatty acid/kg tissue (wet weight), and as weight percentage of the measured fatty acids.

### Estimates of fatty acid desaturases

We have used product/precursor ratios as crude estimates of the formation of OA (AA), catalyzed by desaturase/elongase, i.e. the OA to stearic acid (Stear) ratio for Delta-9 desaturase (OA synthesis), and the AA/LA ratio to estimate Delta-5/6 desaturase and Elongase-5 (AA formation). Furthermore, we have considered whether the relative abundance of OA (AA) might serve as effectors of a feedback regulation. We accordingly studied 3 bivariate associations: a) the OA/Stear ratio vs. the AA/LA ratio; b) % OA vs. the AA/LA ratio; and c) % AA vs. the OA/SA ratio.

### Statistical analysis

On the pooled sample, the relationship between percentages of fatty acids was assessed by correlation (Pearson), and by multiple linear regression. The OA/ AA ratio served as the principal dependent variable and ALA was the independent variable under investigation. We made 4 regression models: Model 1 is without adjustment; Model 2: adjusted for 20:3 n3 + 20:5 n3 + 22:5 n3 + 22:6 n3 ; Model 3 = Model 2 + 18:2 n6 + 18:3 n6 + 20:2 n6 + 20:3 n6; Model 4 = Model 3 + 14:0 + 14:1 c9 + 15:0 + 16:0 + 16:1 c9 + 17:0 + 18:0 + 18:2 c11 + 18:2 n6 + 20:0 + 20:1 n9 + 20:2 n6 + 20:3 n6 + unidentified 18:1 isomers (minor amounts) + diet type. Results are presented in tables, or as scatter plots with the regression line included. SPSS 19.0 was used for the regression analyses and Sigma Plot 2001 for producing the figures. A significance level of 0.05 was accepted.

## Competing interests

The authors declare that they have no competing interests.

## Authors’ contributions

The present study is a spin-off study of a previously published diet trial, conceived and conducted by AH. ATH conceived and designed the present study, analyzed and interpreted the data, and drafted the article. AH contributed substantially to the interpretation of data and revising the article critically for important intellectual content. Both the authors approved the final version to be published.
